# A case report of small bowel occlusion due to Meckel diverticulum causing a life-threatening condition

**DOI:** 10.1016/j.ijscr.2023.108982

**Published:** 2023-10-24

**Authors:** Mohamed Ali Chaouch, Mabrouk Abdelali, Seifeddine Ben Hammouda, Mohamed Zayati, Ahmed Hadj Taieb, Faouzi Noomen

**Affiliations:** a Department of Visceral and Digestive Surgery, Monastir University Hospital, Monastir, Tunisia; b Department of Radiology, Monastir University Hospital, Monastir, Tunisia; c Department of Pathology, Monastir University Hospital, Monastir, Tunisia

**Keywords:** Meckel's diverticulum, Obstruction, case report, complications

## Abstract

**Introduction and importance:**

Meckel's diverticulum is a common congenital abnormality. In this study, we reported a case of small bowel occlusion due to Meckel's diverticulum causing a small bowel obstruction and a life-threatening condition.

**Case presentation:**

28-year-old man complaining of abdominal pain for 3 days. The physical examination found the patient appeared profusely unwell with a blood pressure of 80/40 mmHg. The abdominal examination found abdominal rigidity suggesting peritonitis. The patient's C-reactive protein was 210 mg/l with normal white blood cell count. Consequently, the patient was operated on using a laparotomy. A mechanical bowel obstruction due to a gangrenous Meckel's diverticulum was confirmed during the operation. Meckel's diverticulitis with the tip attached to the ileal mesentery through a band. A section of the band was performed followed by a resection of the Meckel's diverticulum and an ileo-ileal anastomosis. The postoperative follow-up was uneventful.

Case discussion: Meckel's diverticulum results from yolk sac persistence during embryonic development, often remaining asymptomatic. However, it can lead to symptoms like abdominal pain, diarrhoea, and fever when inflamed or infected. Treatment involves surgery (diverticulectomy) for severe cases with complications, with good prognosis but associated surgical risks.

**Conclusion:**

The diverticulum can present a life-threatening condition. The treatment is essentially surgical. This surgery should be performed emergently to enhance the prognosis.

## Highlights

• Meckel's diverticulum arises from yolk sac persistence during embryonic development and is typically asymptomatic in most individuals.
• Symptoms can occur when the diverticulum becomes inflamed or infected.
• Diverticulectomy is the primary treatment with complications, offering a good prognosis but carrying potential surgical risks.

## 1 Introduction

Meckel's diverticulum is a common congenital abnormality of the small intestine that occurs in approximately 2 % of the population [1]. It is a pouch or sac that protrudes from the wall of the small intestine, and it is located in the lower right side of the abdomen, near the ileocecal valve. In most cases, this affection remains asymptomatic. However, Meckel's diverticulum can also cause complications such as intestinal obstruction, bleeding, or perforation [2]. Treatment for Meckel's diverticulum typically involves surgical removal of the diverticulum if it causes symptoms or complications [3]. In this study, performed using SCARE guidelines [4], we reported a case of small bowel occlusion due to Meckel's diverticulum causing a life-threatening condition. The present paper was reported in line with the SCARE guidelines.

## 2 Case presentation

**Fig. 1:**
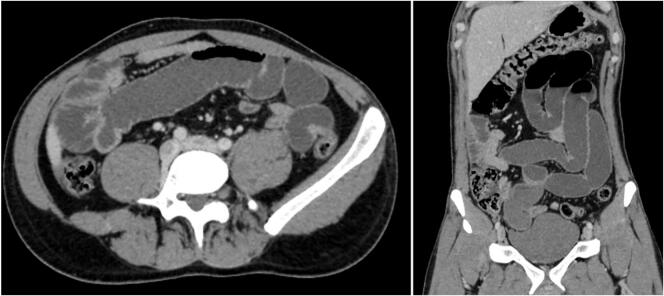
CT scan views suggesting the presence of a tabulated diverticular structure, hanging from the terminal ileum, distended with air-fluid content with a wall that presents a lack of focal enhancement and a focal stricture. The ileal distension described above sits upstream of this structure: the whole evokes a mechanical OIA of the small intestine upstream of a complicated Meckel's diverticulum.

**Fig. 2:**
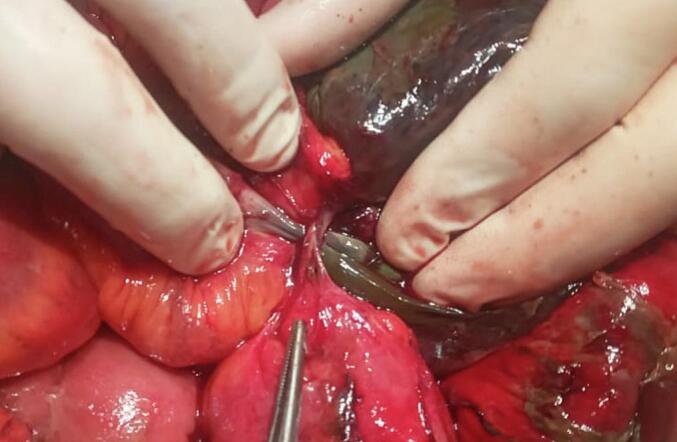
Intraoperative view showing the mechanical small bowel occlusion due the band in the Meckel diverticulum.

**Fig. 3:**
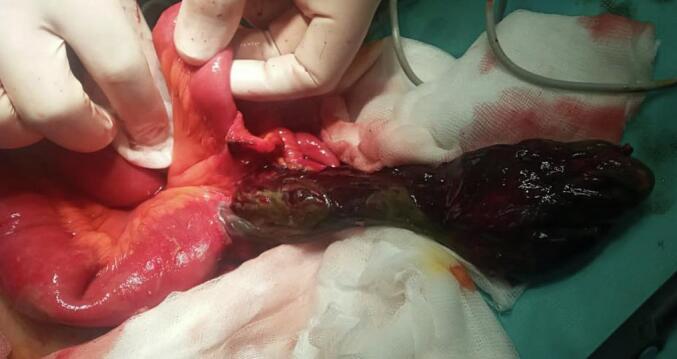
Intraoperative view of the necrotic Meckel diverticulum.

**Fig. 4:**
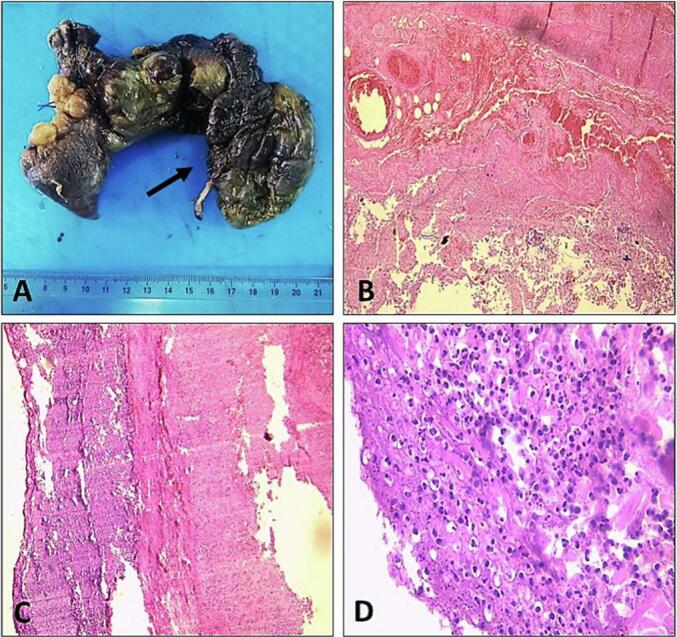
Gross pathology specimen reveals a giant Meckel's diverticulum with dusky and necrotic appearance, which is attached to the small intestine (black arrow). B, C (Hematoxylin-Eosin (HE); ×4): Microscopic findings showing transmural small bowel necrosis, with nonviable mucosa, hemorrhage, edema and congestion of wall. There is no ectopic tissue. D (HE; ×40): Acute inflammatory infiltrate in the serosa of the Meckel's diverticulum.

A 28-year-old man, with no past medical history, presented to the Emergency Department with a 3-day history of abdominal pain and vomiting. The patient reported no other associated symptoms. The patient reported an episode similar to the current one two years ago. This condition was managed conservatively and resulted in a prompt resolution of his symptoms within 24 h of his presentation to the hospital. The patient remained asymptomatic thereafter with no consequent episodes till the current presentation. On the physical examination, the blood pressure was 80/40 mmHg. The abdominal examination found abdominal rigidity suggesting peritonitis. The patient's C-reactive protein was 210 mg/l. To better delineate the aetiology underlying the patient's presentation, an abdominal CT scan was performed and revealed multiple air-fluid levels in the small bowel suggesting a mechanical small bowel obstruction (Fig. 1). The presence of Meckel's diverticulum due to an acute small bowel obstruction was deemed exceedingly plausible. Consequently, the patient was operated on using a laparotomy after putting in a nasogastric tube. During the operation, a mechanical bowel obstruction due to a gangrenous Meckel's diverticulum close to the ileocecal valve was confirmed. Per-operative evaluation divulged Meckel's diverticulitis with the tip attached to the ileal mesentery suggesting a mesodiverticular band (Fig. 2, Fig. 3). A section of the band was performed followed by a resection of the Meckel's diverticulum and an ileo-ileal isoperistaltic side-to-side manual anastomosis. The postoperative follow-up was uneventful and the patient was discharged after 5 days. He was examined after 15 days. The pathological examination of the operative specimen concluded with a giant Meckel's diverticulum (Fig. 4).

## 3 Discussion

Meckel's diverticulum is formed when a portion of the yolk sac fails to disappear during embryonic development. Meckel's diverticulum is a vestigial remnant that arises from the failure of the omphalomesenteric duct to close. The omphalomesenteric duct connects the yolk sac in the embryo to the early foregut through the umbilical cord. The diverticulum may vary in size and shape, and it may contain tissue resembling the stomach lining or pancreas [5]. Most people with Meckel's diverticulum do not experience any symptoms and may not even be aware of the presence of the diverticulum. However, in some cases, the diverticulum can become inflamed or infected, leading to symptoms such as abdominal pain, diarrhoea, and fever. Meckel's diverticulum is typically diagnosed using imaging tests such as ultrasound, CT scan, or MRI [6]. In case of non-emergent surgery, use a technetium-99m (99mTc) pertechnetate scan, also called Meckel scan or nuclear scintigraphy scan which is the investigation of choice to diagnose Meckel's diverticula. Treatment depends on the severity of the symptoms and complications. In mild cases, observation and symptomatic treatment may be sufficient. However, in severe cases, surgery may be necessary to remove the diverticulum and repair any damage to the intestine [7]. The surgical treatment of Meckel's diverticulum depends on the severity of the symptoms and complications. In most cases, surgery is only necessary if the diverticulum is causing significant symptoms or complications, such as inflammation, infection, bleeding, or obstruction of the intestine [3]. In the case of a small bowel occlusion, a mesodiverticular band is generally found. The surgical procedure used to treat Meckel's diverticulum is called a diverticulectomy. During this procedure, the surgeon makes an incision in the lower abdomen, locates the diverticulum, and removes it along with a small portion of the adjacent small intestine. The remaining ends of the intestine are then stitched back together [3]. If the Meckel's diverticulum has caused damage to the intestine, such as perforation or infection, the surgeon may need to perform additional procedures, such as repairing the damaged area or removing a portion of the intestine. During the Meckel's diverticulum resection, we should avoid any digestive tract perforation causing contamination of the peritoneal cavity and we should control the small bowel mesentery to avoid bleeding. In general, the prognosis for patients who undergo surgical treatment for Meckel's diverticulum is good, and most patients can recover fully and return to their normal activities within a few weeks after the procedure [5,8]. However, as with any surgery, there are risks associated with the procedure, including bleeding, infection, and damage to nearby organs or tissues.

## 4 Conclusion

The Meckel diverticulum is often asymptomatic. When it is complicated it can present a life-threatening condition. The treatment is essentially surgical. This surgery should be performed emergently to enhance the prognosis. The final confirmation of the diagnosis remains after the pathological examination of the operative specimen.

## Consent

Written informed consent was obtained from the patient for publication of this case report and accompanying images. A copy of the written consent is available for review by the Editor-in-Chief of this journal on request.

## Ethical approval

Ethical approval is exempt/waived at our institution.

## Funding

This research received no specific grant from the public, commercial, or not-for-profit sectors.

## Author contribution

All the authors participated in the treatment of the patients, writing, and approving the manuscript.

## Guarantor

Mohamed Ali Chaouch, MD.

## Conflict of interest statement

No conflict of interest to disclose.
